# Propofol Attenuates Toxic Oxidative Stress by CCl_4_ in Liver Mitochondria and Blood in Rat

**Published:** 2014

**Authors:** Akram Ranjbar, Mohammad Sharifzadeh, Jamshid Karimi, Heidar Tavilani, Maryam Baeeri, Tavakol Heidary shayesteh, Mohammad Abdollahi

**Affiliations:** a *Department of Toxicology and Pharmacology, School of Pharmacy, Hamadan University of Medical Sciences, Hamadan, Iran.*; b *Faculty of Pharmacy, and Pharmaceutical Sciences Research Center, Tehran University of Medical Sciences, Tehran, Iran.*; c *Department of Biochemistry, School of Medicine, Hamadan University of Medical Sciences, Hamadan, Iran.*

**Keywords:** Propofol, Liver mitochondria, Oxidative stress, Rat, CCl_4_

## Abstract

Anti-oxidant effects of propofol (2, 6-diisopropylphenol) were evaluated agains carbon tetrachloridet CCl_4_ -induced oxidative stress in rat liver. 30 male rats were equally divided in to 6 groups (5 rats each). Group I (control), while Group II was given CCl_4_ (3 mL /Kg/day, IP). Animals of Groups III received only propofol (10 mg/Kg/day, IP). Group IV was given propofol+ CCl_4_. Group V was administered vitamin E (alpha-tocopherol acetate 15 mg/Kg/day, SC) .Animals of Group VII received alpha-tocopherol acetate + CCl_4_ once daily for two weeks. After treatment, blood and liver mitochondria were isolated. Anti-oxidant enzymes activity such as glutathione peroxidase (GPx), superoxide dismutase (SOD) and oxidative stress marker such as reduced glutathione (GSH) and lipid peroxidation (LPO) concentration were measured. Oxidative stress induced with CCl_4_ in liver mitochondria was evident by a significant increase in enzymatic activities of GPx, SOD, and LPO and decreased of GSH and vailability of mitochondria. Propofol and vitamin E restored CCl_4_-induced changes in GSH, GPx, SOD and LPO in blood and liver mitochondria. CCl_4_ decreased viability of mitochondria that was recovered by propofol and vitamin E. It is concluded that oxidative damage is the mechanism of toxicity of CCl_4_ in the mitochondria that can be recovered by propofol comparable to vitamin E.

## Introduction

Propofol (2, 6-diisopropylphenol) is a widely used intravenous sedative-hypnotic agent for both induction/maintenance of anesthesia and sedation of critically ill patients (1) Advantages of this agent over others of similar applications include lower incidence of side effects (2) and better quality of anesthesia (3). Propofol's structure contains a phenolic hydroxyl group and thus resembles that of a-tocopherol (vitamin E), a natural anti-oxidant. As shown by both *in-vitro* and *in-vivo* studies, the anti-oxidant activity of propofol results partly from this phenolic chemical structure (4). Numerous studies have demonstrated anti-oxidant effects of propofol *in-vitro* (5, 6) and *in-vivo* (7), but actions of propofol on different cells are varied and multiple mechanisms may be involved (8). Propofol has been demonstrated to prevent oxidative stress-mediated endothelial cell activation and dysfunction induced by hydrogen peroxide (8, 9) and tumour necrosis factor-a (TNF-a) (10, 11). The anti-oxidant status of a cell determines its susceptibility to oxidative damage, and is usually altered in response to oxidative stress (12). When reactive oxygen species (ROS) generation overwhelms the anti-oxidant defense, the free radicals can interact with endogenous macromolecules and alter cellular function (12). The mitochondrial respiratory chain is the major source of intracellular generation of ROS and at the same time, an important target for the damaging effects of ROS (13).

Carbon tetrachloride (CCl_4_) is known to induce reactive free radicals and induction of cell damage through covalent binding to the membrane proteins (14).

CCl_4_ is converted in to trichloromethyl radical (CCl_3_•) and its derivative peroxy trichloromethyl radical (•OOCCl_3_) by cytochrome P450 in liver microsomes. These free radicals are highly reactive and are capable of reacting with polyunsaturated fatty acids of the membranous system leading to oxidative injuries such as lipid peroxidation (15). The aims of this study were to investigate the anti-oxidative effects of propofol on liver mitochondrial function in rat treated with CCl_4_**.**

## Experimental

*Chemicals*

Tetraethoxypropane (MDA), 2-thiobarbituric acid (TBA), trichloroace tic acid (TCA), n-butanol, propofol , sucrose, ethylenediamine tetra acetic acid (EDTA), Comassie blue, bovine serum album in (BSA), 4,5(dimethylthiazol-2-yl)-2,5-diphenyltetrazolium bromide (MTT), GPx and SOD (Ransel kit, Randox Laboratories Ltd, Crumlin, UK), bioxytech GSH kit (Oxis Research, USA), Comassie blue, bovine serum albumin (BSA), 4,5-(dimethylthiazol-2-yl)-2,5-diphenyltetrazolium bromide (MTT), were used in this study. All other chemicals were obtained from the Sigma.

*Animals and treatments*

Adult male Wistar rats weighing 180–250 g maintained on a 12-hour light/dark cycle with free access to tap water and standard laboratory chow were used. Animals were randomly divided into six groups (n=5) and treated for 2 week intraperitoneally (IP**). **The groups were as follows: control group, propofol group, CCl_4_ group, propofol and CCl_4_ group, vitamin E group, and CCl_4_ and vitamin E group. 

Propofol was administered (10 mg/Kg/day, IP) alone or in combination with CCl_4_ (0.2 mL/Kg /day, IP) and vitamin E as (15 mg/Kg/day, SC) One group of animals received only normal saline and was assigned as control. At the end of the treatment, 24 hours post the last dose of treatment, animals were killed, liver tissue was separated and stored in liquid nitrogen. Blood samples were collected from heart in heparinized tubes and plasma was isolated. The experiments were conducted according to the ethical rules approved by Institutional Review Board (IRB).

*Preparation of liver mitochondria*

The liver was removed and minced with small scissors in a cold manitol solution containing 0.225 M D-manitol, 75 mM sucrose, and 0.2 mM ethylenediaminetetraacetic acid (EDTA). The minced liver (30 g) was gently homogenized in a glass homogenizer with a Teflon pestle and then centrifuged at 700 × *g *for 10 min at 4 ^◦^C at remove nuclei, unbroken cells, and other non-subcellular tissue. The supernatants were centrifuged at 7,000 × *g *for 20 min. These second supernatants were pooled as the crude microsomal fraction and the pale loose upper layer, which was rich in swollen or broken mitochondria, lysosomes, and some microsomes, of sediments was washed away.

The dark packed lower layer (heavy mitochondrial fraction) was resuspended in the manitol solution and recentrifuged twice at 7,000×*g *for 20 min. The heavy mitochondrial sediments were suspended in Tris solution containing 0.05 M Tris-HCl buffer (pH 7.4), 0.25 M sucrose, 20 mM KCl, 2.0 mM MgCl_2_, and 1.0 mM Na_2_HPO_4_ at 4 ^◦^C before assay (16).

*Measurement of Cu/Zn- SOD*

The activity of Cu/Zn- SOD was measured using a commercial kit (Ransod kit, Randox Laboratories Ltd, Crumlin, UK). Measurement of the enzyme was based on the generation of superoxide radicals produced by xanthine and xanthine oxidase and reacted with 2-(4-iodophenyl)-3-(4-nitrofenol) 5-phenyltetrazolium chloride (INT) to form a red formazan dye. The formazan was read at 505 nm. One unit of Cu/Zn- SOD was defined as the amount of enzyme necessary to produce 50% inhibition in the INT reduction rate. 

*Measurement of GPx*

The amount of GPx was determined using a commercially available kit (Ransel kit, Randox Laboratories Ltd, Crumlin, UK) by measuring the rate of oxidation of NADPH at 340 nm. A unit of enzyme was expressed as the amount of enzyme needed to oxidize 1 nmol of NADPH oxidase/minute. 

*Measurement of* *reduced glutathione assay (GSH)*

Level of reduced glutathione (GSH) was measured using colorimetric assay kit. The kit uses 5, 50-dithiobis-2-nitrobenzoic acid (DTNB) and glutathione reductase. The procedure was followed according to manufacturer’s instruction and the levels were quantitated as micromolar GSH based on standard supplied along with the kit. 

*Measurement of lipid peroxidation (LPO)*

The LPO product in tissues was determined by thiobarbituric acid reagent expressed as the extent of malondialdehyde (MDA) productions during an acid heating reaction. Brieﬂy, the samples were diluted by 1.5 mL TCA (20% w/v) was added to 250 μL o f this samples and centrifuged in 3000 g for 10 min. Then, the precipitation was dissolved in sulfuric acid and 1.5 mL of the mixture was added to 1.5 mL of TBA (0.2% w/v). The mixture was then incubated for 1 h in a boiling water bath. Following incubation, 2 m l of n-butanol was added, the solution centrifuged, cooled and the absorption of the supernatant was recorded in 532 nm. The calibration curve of tetraethoxypropane standard solutions was used to determine the concentrations of TBA+MDA adducts in samples (17). 

*Total Protein*

The protein content was quantiﬁed by the method of Bradford. Concentrated Coomassie blue (G250) was diluted in 250 μL distilled water, and then 750 μL of this diluted dye was added to 50 μL of sample. The mixture was incubated at room temperature for 10 min and an absorbance measurement was taken at 595 nm by a spectrophotometer. A standard curve was constructed by using bovine serum albumin ranging between 0.25 and 1 mg/mL (18).

*Mitochondrial viability*

This assay is a quantitative colorimetric method to determine mitochondrial toxicity or viability of liver mitochondria. It utilizes the yellow tetrazolium salt (MTT), which is metabolized by mitochondrial dehydrogenase enzyme from viable cells to yield a purple formazan reaction product that was determined spectrophotometrically at wavelength of 570 nm. The percentage (of control) of mitochondrial viability of each test sample was calculated (19).

*Statistical analysis*

Mean and standard error values were determined for all the parameters and the results were expressed as Mean + SEM. All data were analyzed with SPSS Version 18 employing one-way ANOVA followed by Tukey post hoc test. Duplicate of experiments were performed. Differences between groups was considered significant when P < 0.05. 

## Results

*Lipid peroxidation (LPO)*

In blood: CCl_4_ caused a significant increase in LPO when compared to control (p-value < 0.001). Propofol caused a significant decrease in LPO when compared to CCl_4_ group (p-value < 0.001). Vitamin E reduced LPO when compared to CCl_4_ (p-value < 0.001). Co administration of Vitamin E with CCl_4_ significantly reduced CCl_4_ induced LPO (p-value < 0.001).

**Figure 1:**
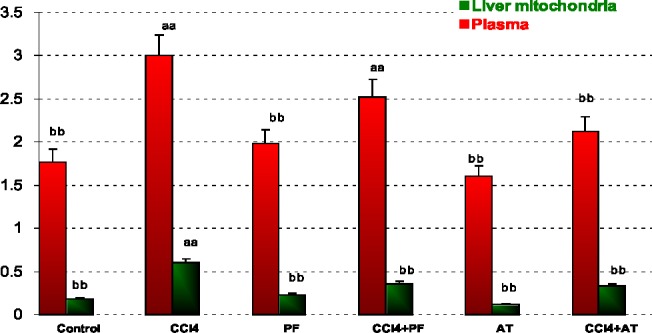
Lipid peroxidation (LPO) in plasma and liver mitochondria of rats. Values are the mean ± SE (n = 5) ^aa^Significantly different from control group at p < .05. ^bb^Significantly different from CCl_4_ group at p < .05. PF, Propofol; AT, (alpha-tocopherol; vitamin E). Duplicate bars from left to right represent plasma and liver mitochondria, respectively

In liver mitochondria: CCl_4_ caused a significant increase in LPO when compared to control (p-value < 0.001). Propofol caused a significant decrease in LPO when compared to CCl_4_ group (p-value < 0.001). Vitamin E reduced LPO when compared to CCl_4_ (p-value < 0.001). Coadministration of propofol or Vitamin E with CCl_4_ significantly reduced CCl_4_ induced LPO (p-value = 0.050 and p-value = 0.010, respectively); Figure 1.

*Superoxide dismutase*

In blood: CCl_4_ caused a significant increase in SOD activity when compared to control (p-value < 0.001). Propofol caused a significant decrease in SOD activity when compared to CCl_4_ group (p-value < 0.001). Vitamin E reduced SOD activity when compared to CCl_4_ (p-value < 0.001).

**Figure 2:**
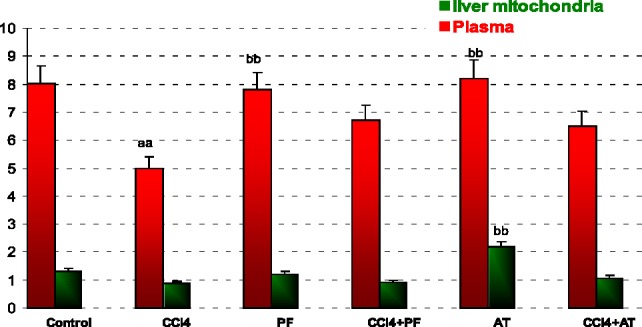
Superoxide dismutase (SOD) activity in plasma and liver mitochondria of rats. Values are the mean ± SE (n = 5) ^aa^Significantly different from control group at ‎p < .05. ^bb^Significantly different from CCl4 group at p < .05. PF, Propofol; AT, (alpha-‎tocopherol; vitamin E). Duplicate bars from left to right represent plasma and liver ‎mitochondria, respectively

In liver mitochondria: Vitamin E reduced SOD activity when compared to CCl_4_ (p-value = 0.014); Figure 2.

*Glutathione peroxidase*

In blood: Vitamin E reduced GPx activity when compared to CCL_4_ (p-value = 0.004). Coadministration of propofol with Vitamin E significantly reduced CCl_4_ induced SOD activity (p-value = 0.003 and p-value = 0.013, respectively).

**Figure 3:**
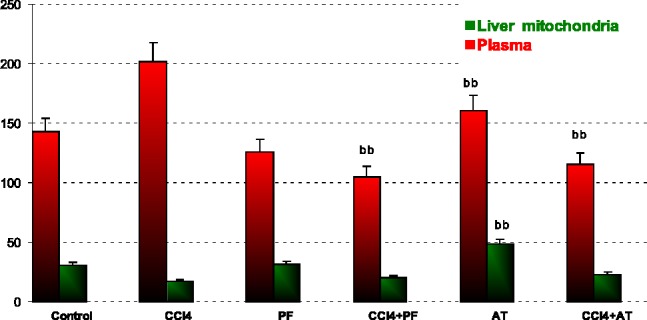
Glutathione peroxidase (GPx) activity in plasma and liver mitochondria of rats. ^aa^Significantly different from control group at p ‎‎< .05. Values are the mean ± SE (n = 5) ^bb^Significantly different from CCl_4_ group at p < .05. PF, Propofol; AT, (alpha-‎tocopherol; vitamin E). Duplicate bars from left to right represent plasma and liver ‎mitochondria, respectively

In liver mitochondria: Vitamin E reduced GPx activity when compared to CCl_4_ (p-value = 0.001); Figure 3.

*Reduced glutathione*

In plasma: Treatment with CCl_4_ decreased GSH as compared to control (p-value = 0.002). Propofol increased GSH as compared to CCl_4_ group (p-value = 0.002). Vitamin E induced GSH when compared to CCl_4_ (p-value = 0.001). 

**Figure 4:**
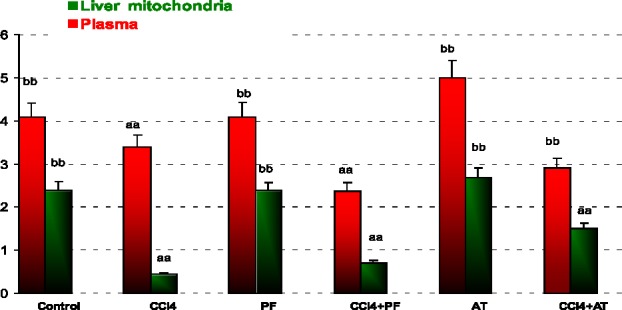
Reduced glutathione (GSH) in plasma and liver mitochondria of rats. ^aa^Significantly different from control group at p ‎‎< .05. Values are the mean ± SE (n = 5) ^bb^Significantly different from CCl_4_ group at p < .05. PF, Propofol; AT, (alpha-‎tocopherol; vitamin E). Duplicate bars from left to right represent plasma and liver ‎mitochondria, respectively

In liver mitochondria: Administration of CCl_4_ decreased GSH in comparison to controls (p- value < 0.001). Treatment with propofol increased GSH as compared to CCl_4_ group (p-value < 0.001). Vitamin E significantly increased GSH level as compared to CCl_4_ group (p-value < 0.001). Coadministration of Vitamin E with CCl_4_ decreased GSH in comparison to propofol (p-value < 0.001); Figure 4.

*MTT cocentration*

**Figure 5:**
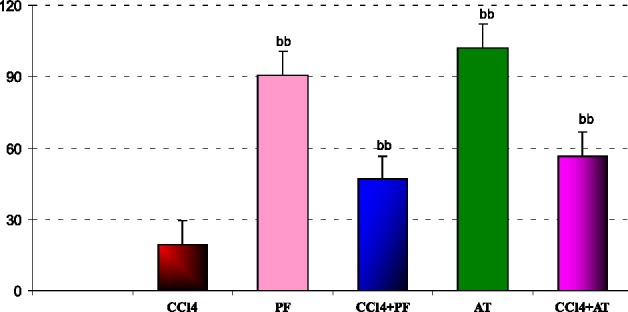
Effect of propofol and CCl4 on percent (of control) mitochondrial viabilities of rat liver. ^aa^Significantly different from control group at p < .05. Values are the mean ± SE (n = 5) ^bb^Significantly different from CCl4 group at p < .05. PF, Propofol; AT,(alpha- tocopherol; vitamin E).

In liver mitochondria: administration of CCl_4_ decreased viability of cells in comparison to Vitamin E group (p-value < 0.001). Treatment with propofol increased viability of cells in comparison to CCl_4_ group ( p-value < 0.001).Coadministration of Vitamin E and propofol with CCl_4_ increased viability of mitochondrial cell when compared to CCL_4_ respectively(p- value < 0.001, p-value = 0.002,); Figure 5. 

## Discussion

Overall results of the present subchronic model of toxicity confirmed potential of CCl_4_ in induction of oxidative injuries and mitochondrial damage. Results indicated that anti-oxidant enzymes GPx, SOD and oxidative marker such as LPO are stimulated in response to exposure to CCl_4_, but their defense was not sufficient to overcome offending free radicals. As observed in the present results, the GSH is also reduced by CCl_4_. Fascinatingly, propofol indicate anti-oxidant properties as well as vitamin E were able to attenuate CCl_4_ induced changes in almost most tested biomarkers.

In other words, CCl_4_ induced oxidative stress by increasing free radicals like induction of cellular damage shown as increase of LPO and reduction of GSH, finally affecting anti-oxidant enzymes activities. The amelioration effect of propofol may be due to chemically similar to endogenous anti-oxidant a-tocopheral (Vitamin E) and theoretically should demonstrate similar properties (20). Numerous studies have demonstrated anti-oxidant effects of propofol *in-vitro* (5, 6, 21) and *in-vivo* (7), but actions of propofol on different cells are varied and multiple mechanisms may be involved. This result indicates reduction of LPO in propofol treatment group induced by CCl_4_. The anti-oxidant effects of propofol may also be due to its ability to attenuate the formation of lipid peroxides (22), to induce the expression of anti-oxidant enzyme heme oxygenase-1 (6), to decrease the expression of nitric oxide synthase (NOS), (23) and to stabilize the mitochondrial membrane (24). Our findings showed that, propofol reduced oxidative biomarkers against CCl_4_ toxicity in plasma and liver mitochondria. We determined that the propofol, mitochondrial toxicity significantly decreased, whereas it increased significantly in liver mitochondria following CCl_4_ administration. We think it is due to anti-oxidant properties of propofol in liver mitochondria. Propofol was also shown to promote mitochondrial function by stabilizing the transmembrane electrical potential (25, 26) and inhibiting mitochondrial permeability transition pore opening (27), both contributing to suppression of mitochondrion-dependent apoptotic signaling (28).These findings indicate that the toxic stress of CCl_4_ and the protective effects of propofol extensively involve mitochondrial viability (Figure 5). The injuries induced by CCl_4_ are resulted from free radicals through lipid peroxidation of cell membranes, reduces anti-oxidant enzyme and anti-oxidant substrates to induce oxidative stress that is an important factor in acute and chronic injuries in various tissues (15). The low levels of LPO in the groups receiving propofol in our study suggest that propofol prevents the lipid peroxidation caused by CCl_4_ (Figure 1).

Oxidative stress may result in overproduction of oxygen free-radical precursors and/or decreased efficiency of the anti-oxidant system. CCl_4_ and oxygen free-radical generation is associated with impaired glutathione metabolism, alterations in the anti-oxidant status (29). However, oxidative stress through generation of reactive oxygen species (ROS) plays an important role in producing liver damage and initiating hepatic fibrogenesis. Oxidative disruption of lipids, proteins, and DNA induces necrosis and apoptosis of hepatocytes, and amplifies the inflammatory response, resulting in initiation of fibrosis. Additionally, ROS stimulate the production of profibrogenic mediators from Kupffer cells and circulating inflammatory cells (30). In present study propofol inhibition of ROS formation, scavenging ROS, or interfering with ROS pathogenic signaling pathways might be the potential ways to protect mitochondrial cells from dysfunction by CCl_4_. While the contribution of Complex III to mitochondrial ROS production in the absence of respiratory inhibitors remains unresolved, two known effects of mitochondrial Ca^+2^ could conceivably promote ROS production at this site. One of the key events that cause mitochondrial injury is an abnormal increase in intracellular Ca^+2^ (31, 32). It is well known that mtPT can be mediated by a concerted action between Ca^2+^ and ROS, where ROS promote the oxidation and crosslinkage of mitochondrial membrane protein thiol groups such as GSH (33). The mechanisms of Ca^+2^-mediated mitochondrial damage involves activation of degradative enzymes, *e.g.,* calpain proteases and phospholipases, or enzymes that generate reactive oxygen species (ROS) capable of oxidatively modifying mitochondrial proteins and lipids (34, 35). Reduced glutathione is a substrate for mitochondrial matrix glutathione peroxidase, an anti-oxidant enzyme that detoxifies H_2_O_2_ and other peroxides (36-38). The results of our present investigation showed that 0.2 mL/Kg CCl_4_ administration in rats caused significant induction in the activity of anti-oxidant enzymes and decreased GSH. GSH is a critical endogenous anti-oxidant involved in cell defense (39). Although GSH tends to be conserved preferentially in mitochondria, the concentration of GSH in mitochondria may be most relevant for the disposal of ROS and cell fate (40-42). The intracellular distribution of GSH, particulary interms of relative amounts of GSH and disulfide (GSSG), invarious compartments remains unclear. It is a evident, however, that GSH is present in cytosolic, mitochondrial and nuclear pools, can distribute to the nucleous under various condition and is involved in the redox regulation of signal transduction (43-45). Decrease in GSH activity during CCl_4_ toxicity might be due to the decreased availability of GSH resulted during the enhanced oxidative damage (46-48). Improvement of blood and liver mitochondrial GSH levels in rats treated with propofol in comparison to CCl_4_ administration further demonstrated the anti-oxidant properties of this drug (Figure 4). This effect is established via activating the glutathione anti-oxidant system (49, 50).

ROS are produced in normal metabolic processes and are inactivated by endogenous anti-oxidant systems (51). The primary ROS produced by mitochondria is superoxide [89]. This highly reactive free radical is extremely short-lived (52-54) and dismutates either spontaneously or with the help of the mitochondrial superoxide dismutase forming the more ROS, H_2_O_2_ (55, 56). According to our opinion, the significant decrease of GPx and SOD activity in Groups Vitamin when compared to CCl_4_ group may be explained with the suppression of physiologic ROS production by anti-oxidant agents (Figure 2, 3).

Recently *in-vitro* studies showed that propofol could effectively suppress apoptotic signaling and prevent apoptotic death of myocardial cells encountering fatal stimuli (6, 57, 58). In experimental hearts, propofol inhibited mitochondrial permeability transition (27) and ameliorated ischemia–reperfusion injury (59-61). In fact, sustained hepatic inflammation provoked by long-term treatment with CCl_4_ is believed to be through NF-κB pathway (62, 63). NF-κB is a transcription factor consisting of p65 and p50 subunits of the Rel protein family that regulates host inflammatory and immune responses by increasing the expression of specific cellular genes (64, 65). Released activated NF-κB complex tranlocates to the nucleus, where it regulates gene expression by binding to κB binding sites (61, 66). 

These findings support the premise that propofol can guard against the sequences of oxidative stress. Hence, the anti-oxidant properties of propofol are involved in its hepatoprotective mechanism. The powerful anti-oxidant activity of propofol has been examined previously in different oxidative stress situations (67). Interestingly, as evidenced by MTT assay, the present data confirm that CCl_4_-induced mitochondrial dysfunction can be improved by propofol. However, the exact molecular mechanisms of a putative protective role of propofol in hepatoprotection should be further investigated.
